# Intrastriatal convection-enhanced delivery results in widespread perivascular distribution in a pre-clinical model

**DOI:** 10.1186/2045-8118-9-2

**Published:** 2012-01-20

**Authors:** Neil U Barua, Alison S Bienemann, Shirley Hesketh, Marcella J Wyatt, Emma Castrique, Seth Love, Steven S Gill

**Affiliations:** 1 Department of Neurosurgery, Frenchay Hospital, Bristol, BS16 1LE, UK; 2 Functional Neurosurgery Research Group, University of Bristol, BS10 5NB, UK; 3 Department of Neuropathology, Frenchay Hospital, Bristol, BS16 1LE, UK

**Keywords:** Convection-enhanced delivery, dextrans, solutes, perivascular, macrophages, nanoparticles

## Abstract

**Background:**

Convection-enhanced delivery (CED), a direct method for drug delivery to the brain through intraparenchymal microcatheters, is a promising strategy for intracerebral pharmacological therapy. By establishing a pressure gradient at the tip of the catheter, drugs can be delivered in uniform concentration throughout a large volume of interstitial fluid. However, the variables affecting perivascular distribution of drugs delivered by CED are not fully understood. The aim of this study was to determine whether the perivascular distribution of solutes delivered by CED into the striatum of rats is affected by the molecular weight of the infused agent, by co-infusion of vasodilator, alteration of infusion rates or use of a ramping regime. We also wanted to make a preliminary comparison of the distribution of solutes with that of nanoparticles.

**Methods:**

We analysed the perivascular distribution of 4, 10, 20, 70, 150 kDa fluorescein-labelled dextran and fluorescent nanoparticles at 10 min and 3 h following CED into rat striatum. We investigated the effect of local vasodilatation, slow infusion rates and ramping on the perivascular distribution of solutes. Co-localisation with perivascular basement membranes and vascular endothelial cells was identified by immunohistochemistry. The uptake of infusates by perivascular macrophages was quantified using stereological methods.

**Results:**

Widespread perivascular distribution and macrophage uptake of fluorescein-labelled dextran was visible 10 min after cessation of CED irrespective of molecular weight. However, a significantly higher proportion of perivascular macrophages had taken up 4, 10 and 20 kDa fluorescein-labelled dextran than 150 kDa dextran (*p *< 0.05, ANOVA). Co-infusion with vasodilator, slow infusion rates and use of a ramping regime did not alter the perivascular distribution. CED of fluorescent nanoparticles indicated that particles co-localise with perivascular basement membranes throughout the striatum but, unlike soluble dextrans, are not taken up by perivascular macrophages after 3 h.

**Conclusions:**

This study suggests that widespread perivascular distribution and interaction with perivascular macrophages is likely to be an inevitable consequence of CED of solutes. The potential consequences of perivascular distribution of therapeutic agents, and in particular cytotoxic chemotherapies, delivered by CED must be carefully considered to ensure safe and effective translation to clinical trials.

## Background

One of the major obstacles to the effective treatment of neurological disorders ranging from brain tumours to neurodegenerative diseases is the blood-brain barrier (BBB), which regulates the passage of molecules from the circulation into the central nervous system. Increasing the dose of systemically administered therapeutics has the potential to increase intracerebral drug delivery. However, in the case of drugs such as chemotherapies, this strategy risks increased systemic side-effects which cannot be tolerated by patients. Consequently, there has been considerable neurosurgical interest in developing methods of direct interstitial drug delivery to the brain in order to bypass the BBB.

Traditionally, intracerebral drug injection techniques rely on diffusion of the therapeutic agent from the site of introduction, significantly limiting the volume of distribution [1]. Furthermore, the clinical utility of intracerebral drug injection may be hampered by tissue damage associated with insertion of cannulae for drug delivery [2]. Convection-enhanced delivery (CED) has emerged as an alternative strategy for intracerebral drug delivery using intraparenchymal microcatheters and has shown significant potential in clinical trials [3,4]. By establishing a pressure gradient at the tip of a very fine catheter, drugs can be delivered in uniform concentration through a much larger volume of interstitial fluid. CED confers several potential advantages over conventional intracerebral injection methods, including the distribution of therapeutic agents throughout large and clinically-relevant brain volumes and avoidance of excess tissue damage. Since the concept of pressure-mediated interstitial drug delivery was first described in 1994 by Bobo *et al*. [5], pre-clinical researchers have focused on maximising the volume of drug distribution. It is now clear that the ability to achieve widespread, predictable and reproducible CED without reflux of infusate, is fundamentally reliant on a range of factors including catheter design, and the physicochemical properties and tissue affinity of the infused agent [2,6-8].

In 2005 Krauze *et al*. [9] reported that CED of liposomes into the putamen of non-human primates caused the liposomes to be distributed within perivascular spaces along branches of the lateral striate and middle cerebral arteries. The authors suggested that the perivascular pathways of the brain might serve as a conduit not only for endogenous molecules (as had been proposed by Szentistvanyi, Weller and others), but also interstitially infused agents [10-12]. A number of studies have demonstrated that tracers injected into the brain parenchyma drain along perivascular spaces in grey matter and the subarachnoid space [11,13,14]. The unidirectional nature and rapidity of solute transport appeared to be driven by arterial pulsations, and perivascular distribution of solutes has been shown to be absent following cardiac arrest [15]. Furthermore the interstitial distribution of therapeutic molecules delivered by CED is significantly restricted by hypotension, consistent with a "perivascular pump" driven by arterial pulsations [16]. Mathematical modelling suggests that the effective perivascular elimination of soluble metabolites and waste products from the brain is fundamentally reliant on the expansile nature of the arterial wall [17,18]. Consequently, the stiffening of arteries which occurs with ageing may be a contributing factor to the failure of Aβ elimination seen in cerebral amyloid angiopathy and Alzheimer's disease [19,20].

Recent analysis of the perivascular pathways of the brain suggests that the perivascular drainage pathway for solutes is distinct from that of particulate materials. Carare *et al*. [15] compared the perivascular drainage of solutes with fluorescent particles injected into the corpus striatum of mice and demonstrated that whilst soluble infusates drained along perivascular basement membranes, particulate infusates expanded a potential space between the glia limitans and outer basement membranes of striatal vessels. Particulate infusates were ingested by resident perivascular macrophages, which have wide ranging roles in the initiation, regulation and propagation of neuroinflammatory processes [21-23].

A number of clinical trials of CED of soluble therapeutic agents have been reported in recent years, including chemotherapies for malignant glioma (topotecan, traberdersan, nimustine) and glial cell line-derived neurotrophic factor (GDNF) for Parkinson's disease [3,4,24,25]. It has been postulated that perivascular distribution might result in the immune response seen in some patients receiving intraputaminal delivery of GDNF, as a consequence of interaction with resident perivascular immune cells [9]. The aim of this study was to determine whether the perivascular distribution of solutes (fluorescein-labelled dextrans) delivered by CED into the striatum of rats is affected by the molecular weight of the infused agent, by co-infusion of vasodilator (to reduce local vascular pulsation), alteration of infusion rates or use of a ramping regime. We also wanted to make a preliminary comparison of the distribution of solutes with that of nanoparticles after CED. The potential implications of the widespread perivascular distribution of solutes for future clinical trials are also considered.

## Methods

### Animal procedures

All *in vivo *studies were performed in accordance with University of Bristol animal care policies and with the authority of appropriate UK Home Office licences. Adult male Wistar rats (Charles River, Margate, UK, 225 to 275 g) were anaesthetised with intraperitoneal ketamine (Ketaset; 60 mg/kg, Pfizer Animal Health, Sandwich, UK) and medetomidine (Dormitor; 0.4 mg/kg, Pfizer), and then placed in a stereotactic frame (Stoelting, Illinois, USA). A midline skin incision was made from glabella to occiput to expose bregma. Bilateral burr holes were drilled using a 2 mm dental drill. All CED procedures were performed using a custom-made catheter with an outer diameter of 0.22 mm and inner diameter of 0.15 mm, composed of fused silica. The cannula was attached to a 1 ml syringe (Hamilton, Bonaduz, Switzerland) connected to a rate-controlled microinfusion pump (World Precision Instruments Inc., FL, USA) and the tip placed at stereotactic co-ordinates derived from the Paxinos and Watson stereotactic rat brain atlas (0.75 mm anterior and 3 mm lateral to bregma, depth 4.5 mm), in order to target the striatum [26]. A total of 6 infusions (bilateral striatal procedures in 3 animals) were performed for each molecular weight of dextran per time point (10 min and 3 h). Similarly, a total of 6 infusions of 20 nm fluospheres were performed per time point.

### CED of fluorescein-labelled dextran and fluorescent nanoparticles

Solutions of 4, 10, 20, 70 or 150 kDa fluorescein-labelled dextran (Sigma-Aldrich, Gillingham, UK) in artificial cerebrospinal fluid (Torbay Pharmaceuticals, Torbay, UK) at a concentration of 1 mg/ml were used for solute infusions. Suspensions of 2% carboxylate-modified fluorescent nanoparticles of 20 nm diameter (Invitrogen, Paisley, UK) were used for particulate infusions. Carboxylate-modified nanospheres were chosen for their relative hydrophilicity and net negative charge (characteristics which are known to confer optimum interstitial distribution characteristics) [6]. Nanoparticles with 20 nm diameter were selected to allow comparison to 150 kDa dextran which has an estimated hydrodynamic diameter of between 16 and 21 nm [27].

A total volume of 5 μl was delivered into the striatum. CED procedures were performed at an infusion rate of 2.5 μl/min unless otherwise stated. On completion of CED the cannula was left *in situ *for 5 min to minimise reflux, then withdrawn at a rate of 1 mm/min. The wound was closed with 4/0 Vicryl, and a dose of intramuscular buprenorphine was administered (30 μg/kg). The anaesthetic was reversed with 0.1 mg/kg i.p. atipamezole hydrochloride (Pfizer, Kent, UK) in animals recovered for 3 h following infusion. Rats were euthanised by anaesthetic overdose with an intraperitoneal injection of 1 ml pentobarbital (Euthatal; Merial Animal Health, Harlow, UK), either immediately after removal of the CED catheter (10 min following termination of CED) or 3 h after infusion. The 10-min time point represents the earliest possible time following slow withdrawal of the CED cannula (which is required to reduce reflux of infusate). The 3 h time point was selected as this has previously been shown to be the earliest time at which perivascular macrophage uptake of tracers is detectable in studies investigating injection of solutes into mouse striatum [15]. Following death, animals were transcardially perfused with 4% paraformaldehyde. Brains were removed and placed in 4% paraformaldehyde for 24 h, then cryoprotected in 30% sucrose.

### Immunohistochemistry

Serial cryostat sections of brain, 25 μm thick, were immunolabelled to identify perivascular tissue elements in the brain following injection of dextran. Vascular endothelial cells were labelled with RECA1 monoclonal antibody (Abcam, Cambridge, UK, 1:300), the laminin component of vascular basement membranes with a polyclonal antibody (Sigma-Aldrich, 1:300) and perivascular macrophages with ED1 monoclonal antibody (Abcam, 1:300). Leptomeningeal macrophages were also identified by staining with ED1 monoclonal antibody. Smooth muscle cells in the walls of arteries were identified by staining for α-smooth muscle actin monoclonal antibody (Sigma-Aldrich, 1:200). For fluorescence immunohistochemistry, Cy3 (Jackson Laboratories, CA, USA) or Alexa Fluor 350 (Invitrogen) species-specific secondary antibodies were used at a dilution of 1:200. All incubations with primary antibodies were for 18 h at 4°C, and 2 h at room temperature for secondary antibodies.

### Co-administration of nimodipine

To investigate the effect of local vasodilatation on the perivascular distribution of solutes delivered into striatum by CED, solutions of 10 and 150 kDa fluorescein-labelled dextran were co-infused with the calcium channel antagonist nimodipine (Bayer Pharmaceutical Corporation, Newbury, UK). An initial pilot study was performed in order to identify the concentration of nimodipine required to achieve maximal vasodilatation of arteries within the striatum. Fluorescein-labelled dextran (10 kDa) was co-infused with nimodipine at concentrations of 0.25, 0.5, 1 and 2 mg/ml using the CED parameters described above. Bilateral infusions of each nimodipine concentration were performed in a single animal. Animals were terminated at 10 min following CED and transcardially perfusion- fixed with 4% paraformaldehyde. Arteries were identified by staining for α-smooth muscle actin in 3 striatal sections at 100 μm intervals. The maximal diameter of smooth muscle actin-stained blood vessels identified within these sections was measured and averaged using Stereoinvestigator software (Microbrightfield, Vermont, USA).

### Investigation of flow rates and ramping regime

To assess whether the infusion flow rate influenced the perivascular distribution, 10 kDa fluorescein-labelled dextran was infused at either 1 or 2.5 μl/min. The effect of ramping (slow stepwise increases in infusion rate) on the extent of perivascular distribution was also investigated by performing infusions of 10 kDa dextran using the following ramping regime: 0.5 μl/min for 2 min, 1 μl/min for 1 min, 2.5 μl/min for 30 sec, 5 μl/min until completion.

### Imaging and analysis of distribution

**Figure 1:**
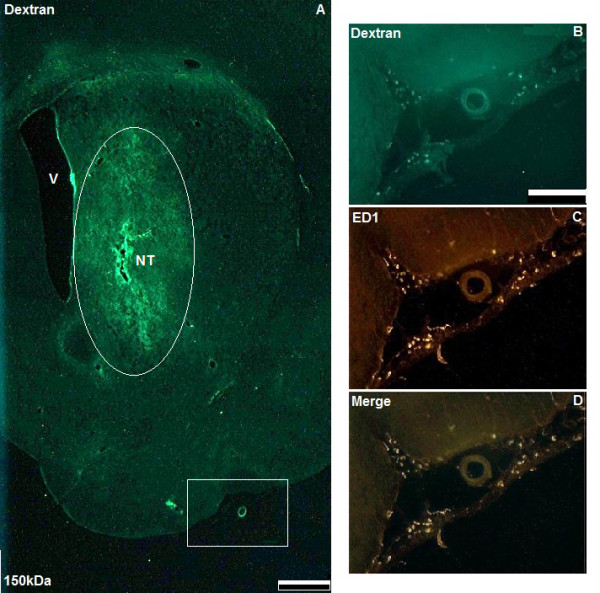
**Widespread interstitial distribution of dextrans and co-localisation with leptomeningeal macrophages following CED**. Figure A shows a low power micrograph of a coronal section through the right striatum of a rat prior to ED1 immunostaining (scale bar = 1 mm). Labelled are the right lateral ventricle (V), needle track (NT) and interstitial distribution of 150 kDa dextran at 3 h following CED (circled). High power micrographs of the area within the white box in Figure A illustrate co-localisation of 150 kDa dextran (green) with ED1-positive leptomeningeal macrophages (red) at 3 h (B, C and D, scale bar = 100 μm).

Fluorescent imaging was performed using a Leica DM5500 microscope (Leica Microsystems, Milton Keynes, UK) and digital camera (CX9000 Microbrightfield). Stereological counts of perivascular macrophages were undertaken using Stereoinvestigator software (Microbrightfield), on sections immunostained for ED1. Co-localisation of fluorescent dextran with perivascular macrophages was quantified in 9 high power fields per striatum (× 20 magnification), on 3 striatal sections at 100 μm intervals, from 3 animals per time point for each dextran molecular weight. A representative image of a striatal section used for analysis following CED of 150 kDa dextran is shown in Figure 1A. Cell counts from 3 striata were then averaged for statistical analysis.

The maximum antero-posterior interstitial spread of each molecular weight of dextran from the injection site was estimated from the number of serial sections demonstrating fluorescence. A qualitative assessment of the distribution of 4, 10, 20, 70 and 150 kDa fluorescein-labelled dextran was also performed by determining whether dextrans were taken up by ED1-positive leptomeningeal macrophages at each time point. Leptomeningeal macrophages were differentiated from perivascular macrophages by their location in the arachnoid membranes on the surface of the brain (Figures 1B, C and 1D).

### Statistical analysis

Statistical analysis was performed using Graphpad Prism statistical software (Graphpad, CA, USA). One-way analysis of variance (ANOVA) was performed with Bonferroni adjustment to compare co-localisation of fluorescein-labelled dextran with perivascular macrophages. Results with *p *< 0.05 were considered significant.

## Results

### Convection enhanced delivery of solutes resulted in widespread perivascular distribution irrespective of molecular weight

**Figure 2:**
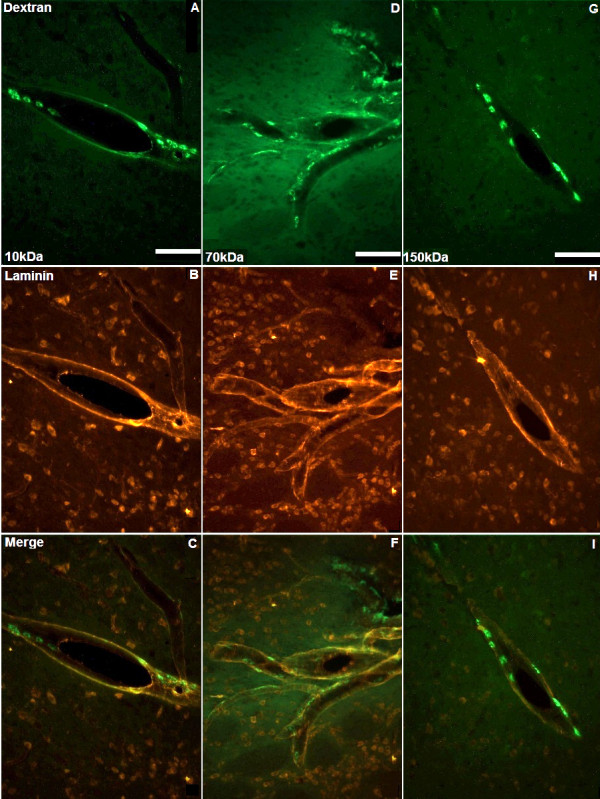
**Co-localisation of solutes with basement membranes**. Ten minutes after CED, 10, 70 and 150 kDa dextrans (green) were widely distributed throughout the perivascular basement membranes of the the striatum (A, D and G). Representative images of perivascular membranes immunostained for laminin (red) are shown in B, E and H. Figures C, F and I represent merged images. Scale bar = 100 μm.

**Figure 3:**
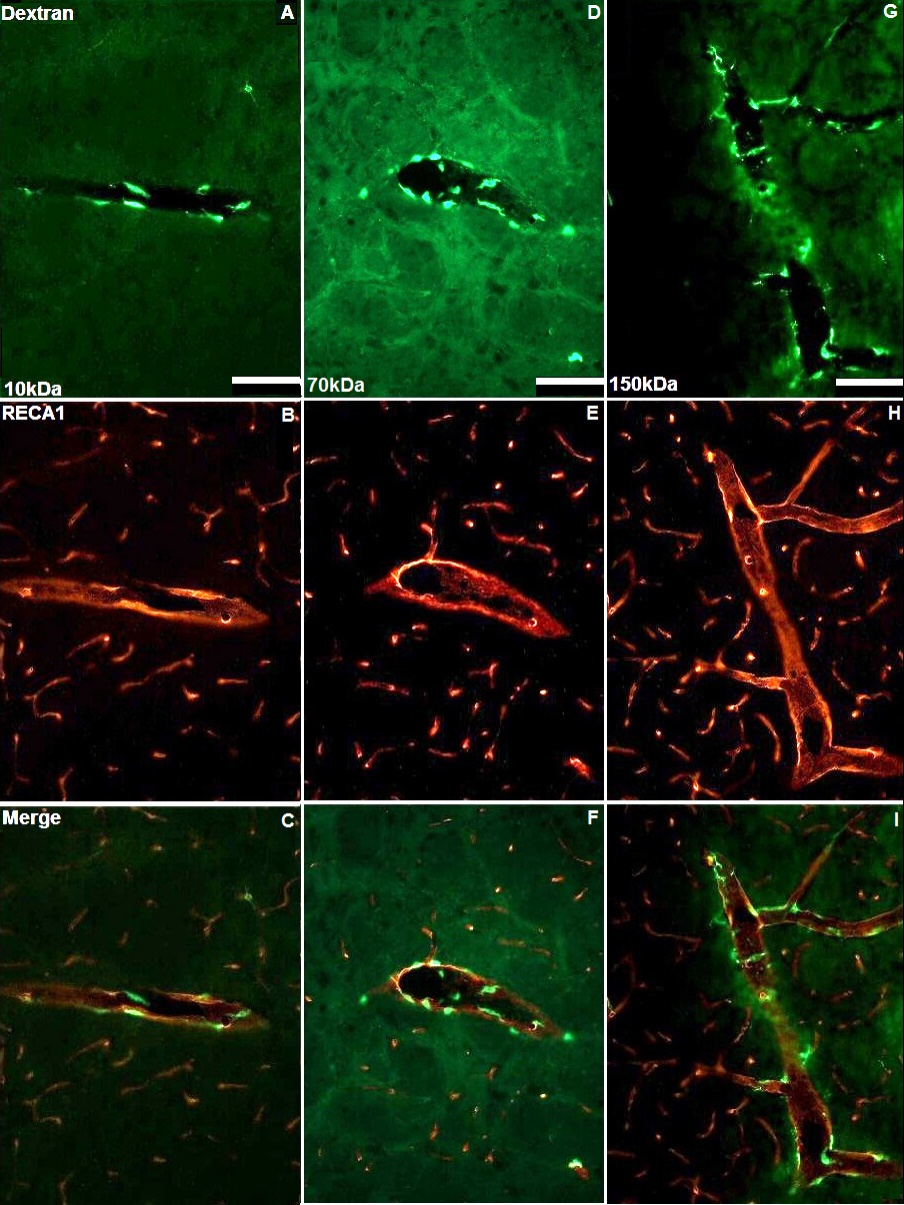
**Perivascular distribution of solutes external to vascular endothelium**. Ten minutes after CED 10, 70 and 150 kDa dextrans (green) were seen in the perivascular spaces external to the vascular endothelium (A, D and G). Representative images of vascular endothelial cells immunostained for RECA1 (red) are shown in B, E and H. Figures C, F and I represent merged images. Scale bar = 100 μm.

**Figure 4:**
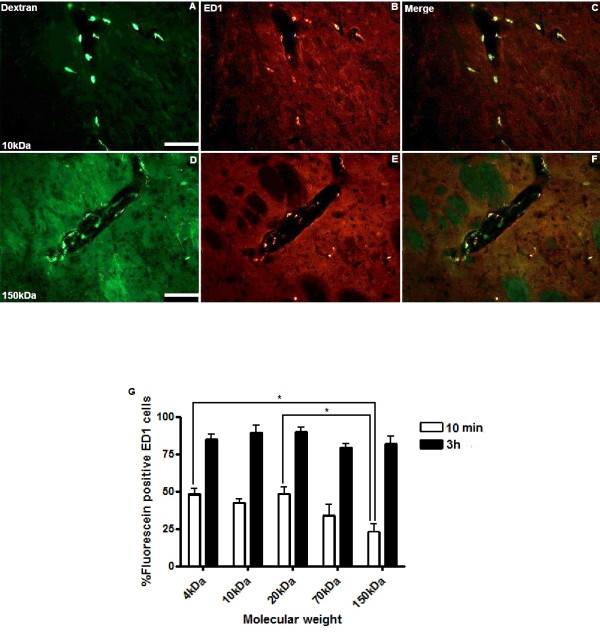
**Co-localisation of fluorescein-labelled dextran with ED1-positive perivascular macrophages**. Ten minutes after CED there was widespread co-localisation of fluorescein-labelled dextran (green) with perivascular macrophages immunostained for ED1 (red). Representative images from animals infused with 10 and 150 kDa dextran are shown in A-F. Scale bar = 100 μm. Stereological cell counts demonstrated significantly higher co-localisation of 4, 10 and 20 kDa dextrans than 150 kDa dextran, 10 min after CED. For 4 and 20 kDa dextran, the increase in co-localisation was highly significant (*p < 0.01). However by 3 h there were no significant differences in uptake (figure G). Each histogram bar represents mean cell counts from 3 striata +/- S.D.

Interstitial and perivascular distribution, as well as perivascular macrophage uptake of 4, 10, 20, 70 and 150 kDa fluorescein-labelled dextran was visible 10 min after cessation of CED (Figures 1, 2 and 3). At this time point, a significantly higher proportion of ED1-positive perivascular macrophages had taken up 4, 10 or 20 kDa fluorescein-labelled dextran than 150 kDa dextran (*p *< 0.05). For 4 and 20 kDa dextran, the increase in co-localisation was highly significant (*p *< 0.01). However, by 3 h there was no significant difference in perivascular macrophage uptake between dextrans of any molecular weight (Figures 4A-F and 4G).

At 10 min following CED, the maximum interstitial spread of all molecular weights of dextran was greater than 2000 μm from the striatal injection site in the antero-posterior plane, as evidenced by the number of serial sections demonstrating fluorescence. At this early time point, all molecular weights of dextran were seen to co-localise with perivascular macrophages within the striatum. Co-localisation of 4, 10 and 20 kDa dextran with ED1-positive leptomeningeal macrophages was also visible at the 10 min time-point. In contrast, co-localisation of 70 and 150 kDa dextrans with leptomeningeal macrophages was only visible at 3 h. By 3 h following CED, 4, 10 and 20 kDa dextran had been cleared from the parenchyma, whilst 70 and 150 kDa dextrans remained visible in the interstitial spaces of the striatum. A summary of the locations of each dextran at 10 min and 3 h following CED is shown in table 1.

**Table 1:** Distribution of dextran following CED into rat striatum

Molecular weight	4 kDa		10 kDa		20 kDa		70 kDa		150 kDa	
**Time point**	**10 min**	**3 h**	**10 min**	**3 h**	**10 min**	**3 h**	**10 min**	**3 h**	**10 min**	**3 h**

Interstitial spaces	+	-	+	-	+	-	+	+	+	+

Perivascular macrophages	+	+	+	+	+	+	+	+	+	+

Leptomeningeal macrophages	+	+	+	+	+	+	-	+	-	+

**+ **present, **- **absent

### Co-infusion with nimodipine did not reduce perivascular distribution

**Figure 5:**
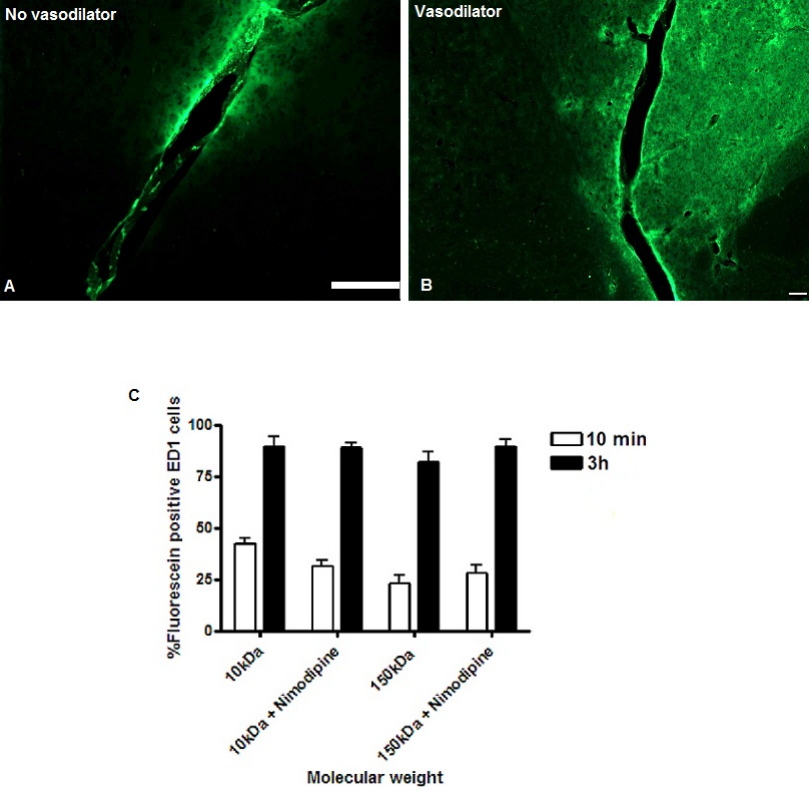
**Co-infusion of 10 and 150 kDa dextran with nimodipine**. Co-infusion of the vasodilator nimodipine did not prevent perivascular spread of solutes. Representative images at 10 min following CED of 150 kDa dextran (green) demonstrating widespread interstitial and perivascular solute are shown in figures A and B. Stereological comparisons of ED1 perivascular macrophage uptake of 10 and 150 kDa dextran with and without nimodipine did not reveal any statistically significant differences (figure C). Each histogram bar represents mean cell counts from 3 striata +/- S.D. Scale bars in both A and B = 100 μm.

The pilot study demonstrated that co-infusion of nimopidine resulted in increasing arterial vasodilation at concentrations of 0.25, 0.5 and 1 mg/ml. However, no additional increase in maximal arterial diameter was demonstrated with a dose of 2 mg/ml. Subsequent CED procedures were therefore performed at a nimopidine concentration of 1 mg/ml. Widespread perivascular distribution and uptake by perivascular macrophages of 10 and 150 kDa dextran was visible despite obvious local vasodilatation (Figure 5A and 5B). There was no significant difference in uptake of either 10 or 150 kDa dextran by perivascular macrophages when the dextrans were infused with or without nimodipine (Figure 5C).

### Slow infusion rates and ramping did not prevent perivascular distribution

**Figure 6:**
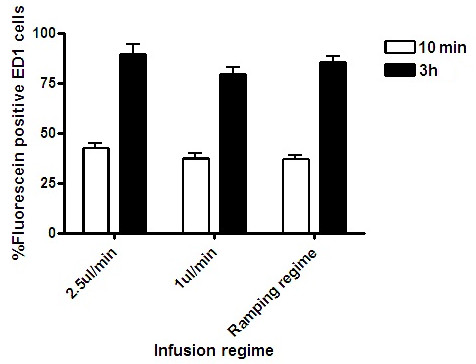
**CED using slow infusion rates and ramping regime**. Slow infusion of 10 kDa dextran at 1 μl/min or use of a ramping regime did not prevent perivascular distribution. Stereological comparisons revealed no significant difference in uptake of 10 kDa Dextran by ED1-positive perivascular macrophages when infused at 1 or 2.5 μl/min or when a ramping regime was used. Each histogram bar represents mean cell counts from 3 striata +/- S.D.

Widespread perivascular distribution of 10 kDa dextran was visible 10 min after cessation of CED whether the dextran was infused at 1 or 2.5 μl/min. Use of a ramping regime did not prevent perivascular distribution. No significant difference in uptake by perivascular ED1-positive cells was demonstrated when 10 kDa dextran was infused at 1 or 2.5 μl/min, or when a ramping regime was used (Figure 6).

### CED of fluorescent nanoparticles

**Figure 7:**
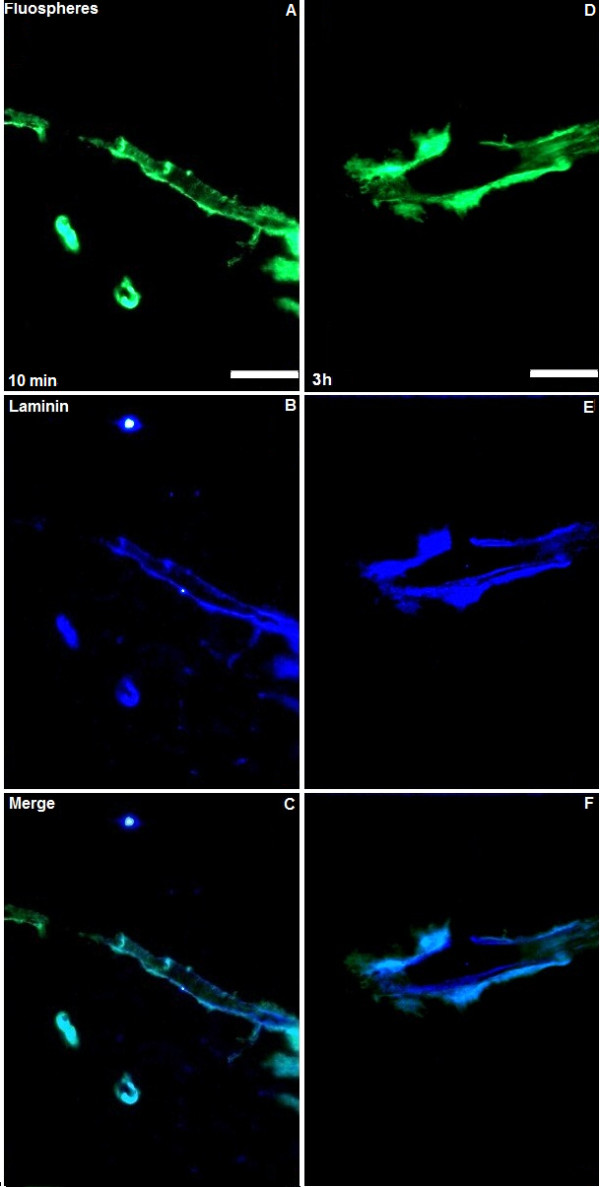
**CED of 20 nm fluorescent particles**. Ten minutes following CED, 20 nm fluospheres (green, figure A) were seen to co-localise with perivascular basement membranes immunostained for laminin (blue, figure B). Appearances at 3 h following CED showed similar co-localisation without evidence of uptake of fluospheres by perivascular macrophages (figures D and E). Figures C and F represent merged images. Scale bar = 100 μm.

Our preliminary studies of CED of 20 nm fluorescent nanoparticles indicate that particles of this size co-localise with perivascular basement membranes throughout the striatum by 10 min following CED but, unlike soluble dextrans, are not taken up by perivascular macrophages at either 10 min or 3 h (Figure 7).

## Discussion

Convection-enhanced delivery has emerged as a promising technique for direct drug delivery to the brain, bypassing the blood brain barrier and enabling distribution of therapeutic agents throughout large volumes of brain parenchyma. In this study we sought to determine whether perivascular distribution is likely to be an inevitable consequence of CED of solutes, and also to determine whether the extent of perivascular distribution is affected by the molecular weight of the solute, co-infusion of vasodilator, the rate of infusion and use of a ramping regime.

Whilst the evidence for the mechanisms which drive perivascular fluid flow in the brain remains controversial, drainage of endogenous solutes along perivascular basement membranes is thought to be a consequence of bulk flow rather than simple diffusion [28]. A number of studies involving injection of tracers into the brain have demonstrated rapid and widespread distribution throughout the perivascular spaces consistent with bulk flow [14,29,11]. Consequently, the molecular weight of solutes might not be expected to affect the rate of perivascular drainage. In this study, significantly more 4, 10 and 20 kDa dextran than 150 kDa dextran had been taken up by perivascular macrophages 10 min following CED. Furthermore, co-localisation of 70 and 150 kDa dextran with leptomeningeal macrophages was only visible at 3 h. It seems most likely that the smaller molecular weight dextrans are simply taken up more efficiently by the macrophages, but we cannot exclude the possibility that the difference reflects an influence of the molecular weight of the solute on the rate of perivascular spread after CED, and consequently the timing of uptake by perivascular macrophages. By 3 h there was no significant difference in the extent of perivascular macrophage uptake of solutes of any molecular weight, perhaps because bulk flow takes over as the predominant driving force behind perivascular drainage once the pressure gradient is removed.

We also sought to determine whether local vasodilatation might reduce the extent of or prevent perivascular distribution of solutes. In a study incorporating mathematical modelling of perivascular drainage, Schley *et al*. [17] postulated that changes in the calibre of blood vessels during each pulse cycle might alter the width of the perivascular spaces thereby providing the motive force for perivascular drainage. In this model, perivascular drainage occurs mainly during periods of vasoconstriction, as a reduction in vessel calibre acts to "open" the perivascular spaces. The authors suggested that a reduction in pulse amplitude, as occurs in ageing, slows the clearance of endogenous solutes (such as Aβ peptide, resulting in perivascular deposition and cerebral amyloid angiopathy). In this study we induced local vasodilatation by co-infusing with nimodipine. By causing prolonged smooth muscle relaxation, we sought to determine whether co-infusion of nimodipine might reduce the perivascular distribution of solutes by preventing vasoconstriction in the period immediately following CED. However, this strategy did not prevent extensive perivascular spread or reduce the amount of 10 or 150 kDa dextran taken up by perivascular macrophages 10 min or 3 h following CED.

The use of slow stepwise increases in infusion rate (ramping) has been investigated in pre-clinical studies, with the aim of increasing the volume of distribution of drugs delivered by CED and to mitigate reflux [30]. Although the exact mechanisms by which ramping promotes interstitial drug distribution over reflux are not fully understood, we can hypothesise that slow stepwise increases in infusion rate might act to mechanically increase the effective pore size of the interstitial spaces of the brain. By increasing the extracellular porosity of the brain parenchyma, ramping might act to reduce the resistance to fluid flow within the interstitial spaces. In our study, there was no difference in perivascular spread or the amount of uptake of 10 kDa dextran by perivascular macrophages when the infusion rate was varied from 1 to 2.5 μl/min, or gradually increased from 0.5 to 5 μl/min. For CED to be effectively translated to clinical trials, it will be necessary to use flow rates within this range for therapeutic agents to be distributed through clinically relevant volumes of brain tissue over a time period which is acceptable to patients [31]. In our forthcoming clinical trial of intraputaminal CED of GDNF for Parkinson's disease, we intend to use flow rates of up to 5 μl/min in order to limit the period of infusion to approximately two hours.

This study suggests that widespread perivascular distribution and interaction with perivascular macrophages is likely to be an inevitable consequence of CED of solutes irrespective of their molecular weight, co-infusion with vasodilators, and use of either slow infusion rates or ramping regimes. Convection-enhanced delivery of viral vectors has previously been shown to result in transfection of perivascular macrophages, suggesting that the interaction with perivascular macrophages seen in this study is not a property of dextrans alone [31]. The recent application of advances in nanoparticle technology to CED raises the question of whether this conclusion is applicable to particulate infusates [32]. Our preliminary studies suggest that whilst CED of 20 nm nanoparticles also results in perivascular distribution, their interaction with perivascular macrophages may differ from solutes. Further studies are needed to investigate the influence of nanoparticle size and charge in greater detail.

There is evidence from pre-clinical studies in both rats and mice that the perivascular spaces of the brain are under immune surveillance by cells of monocyte/macrophage lineage which undergo continuous turn-over [33,34]. These perivascular immune cells have been implicated in neuroinflammatory processes related to cerebral amyloid angiopathy (CAA), Alzheimer's disease (AD) and multiple sclerosis [35-37]. Hawkes *et al*. demonstrated that depletion of perivascular macrophages in a transgenic mouse model of AD resulted in elevation of vascular Aβ level [35]. For disease processes involving the perivascular pathways such as CAA and AD, convection-enhanced delivery might offer a method of directing therapeutic agents to both interstitial and perivascular spaces of the brain.

Conversely, perivascular distribution of some therapies delivered by CED might be undesirable in clinical trials for a number of reasons. Uptake of therapeutic proteins by perivascular macrophages might elicit a neutralising or damaging immune response (which may have contributed to the lack of efficacy seen in clinical trials of GDNF delivery to the putamen [9]). The perivascular drainage of drugs delivered into deep grey matter structures might also result in distribution into the subarachnoid space. In a clinical context, this could result in unwanted side-effects such as nerve root irritation. Furthermore, widespread perivascular distribution of cytotoxic chemotherapies might significantly reduce (or eliminate) the resident perivascular immune cell population, resulting in impaired handling of toxic metabolites and dampening of the innate immune response to tumour cells [35].

## Conclusions

This study suggests that CED of soluble pharmacological agents is likely to result in perivascular distribution and interaction with perivascular macrophages, irrespective of the molecular weight of the therapeutic agent or the infusion regime used. The potential consequences of widespread perivascular distribution of therapeutic agents, and in particular cytotoxic chemotherapies, delivered by CED must be carefully considered to ensure safe and effective translation to clinical trials.

## Competing interests

The authors declare that they have no competing interests.

## Authors' contributions

NB, SL and SG conceived the design of the study. NB, AB, SH, MW and EM carried out the experiments. NB, SL and SG have written the manuscript. All authors have read and approved the final version of this manuscript.
